# α-Fetoprotein contributes to the malignant biological properties of AFP-producing gastric cancer

**DOI:** 10.1515/biol-2022-0476

**Published:** 2023-08-10

**Authors:** Xiang Mao, Jun Wang, Fen Luo

**Affiliations:** Department of General Surgery, Huashan Hospital, Shanghai, 200040, China; Department of General Surgery, Huashan Hospital, No. 12, Middle Urumqi Road, Shanghai, 200040, China

**Keywords:** α-fetoprotein, α-fetoprotein-producing gastric cancer, c-Met, liver metastasis of gastric cancer

## Abstract

This study aimed to investigate whether α-fetoprotein (AFP) could affect the malignant behavior of AFP-producing gastric cancer (AFP-GC) and to explore the relationship between AFP and mesenchymal–epithelial transition factor (c-Met) in AFP-GC. In this study, 23 patients with AFP-GC (AFP[+]) and 18 patients with common gastric cancer (AFP[−]) were evaluated for the c-Met expression using immunohistochemical analysis. The AFP-GC cell line, GCIY, was used. The AFP endoribonuclease-prepared small interfering RNA (siRNA) and eukaryotic AFP overexpression vector were used to increase/knockdown the expression of AFP. Afterward, the c-Met expression was evaluated by polymerase chain reaction and western blot. The proliferation, migration, and invasion of GCIY cells were estimated before and after the AFP overexpression/knockdown. The c-Met expression in both groups was the same (*p* > 0.05), and AFP[+] group had a higher positive incidence of the c-Met expression than the AFP[−] group (*p* < 0.01). Furthermore, the c-Met expression frequency was decreased by AFP knockdown and increased by AFP overexpression (*p* < 0.01). The cell counting kit-8 cell proliferation assay, cell invasion, and migration assays confirmed that the AFP could affect the malignant biological behavior of AFP-GC. These findings suggest that AFP contributes to the malignant biological properties of AFP-GC and the high expression of c-Met in AFP-GC.

## 1 Introduction

α-Fetoprotein (AFP) was identified in 1956 for the first time in a human fetus. AFP was synthesized in the fetus’s liver by the sixth week of conception [1,2]. AFP-producing gastric cancer (AFP-GC) is a distinct histological type of gastric adenocarcinoma, characterized mainly by positive immunoreactivity to AFP and hepatoid differentiation [3,4,5,6,7]. AFP-GC has been categorized as a unique subtype of gastric cancer (GC) [8]. Plenty of attention for further studies has been gained in the last two decades due to the lack of adequate studies on AFP-GC’s clinicopathologic features and prognosis [9]. AFP-GC is a highly malignant type and metastatic compared to the typical GC. However, the association mechanism between excessive malignancy and AFP production is not yet well-defined [10].

Generally, AFP-positive GC had more aggressive behavior than the AFP-negative GC [9]. Even though serum AFP levels are increased in patients with AFP-GC, its incidence may be recurrent without re-elevation of the level of serum AFP [11]. Chang et al. revealed that AFP-producing early GC has the same propensity for liver metastasis as the AFP-producing advanced GC [12]. AFP-GC is associated with high lymphatic metastasis, venous invasion of the gastric wall, and liver metastasis. Recently, AFP-GC was also reported with nonbiliary pancreatitis [13]. The survival rate for patients with AFP-GC is significantly poorer than for patients with other types of GC [3]. AFP-GC has a high degree of malignancy and metastasis frequency [14]. The genetic features of the disease and the essential genes associated with AFP-GC development have not been definitively identified [15]. In addition, AFP-GC has a poor prognosis, but the molecular mechanisms that cause the poor prognosis have not yet been revealed.

Hepatocyte growth factor (HGF) is a pleiotropic cytokine composed of an α-chain and a β-chain [16]. HGF and its receptor c-Met are involved in cancer cells’ progression to malignant invasive phenotypes and the development of distant metastases [17]. According to a study, AFP-GC is associated with a higher expression of c-Met than AFP-negative GC [18]. We designed this study to compare the expression of c-Met in AFP-GC and typical GC. In addition, to explore whether AFP can affect the c-Met expression and malignity in AFP-GC.

## 2 Materials and methods

### 2.1 Study population

A total of 248 patients with GC were admitted for surgery at the Department of General Surgery, Huashan Hospital, Fudan University, China. A total of 28 patients had elevated preoperative serum AFP levels (AFP > 10 ng/mL). AFP was detected in GC cells by immunohistochemical staining in 23 of these 28 patients composed of the AFP-GC group (AFP[+]). Other 23 patients with GC and normal serum AFP levels were selected at random for comparison, and samples of the correspondent GC were tested for AFP immunoreactivity after surgical removal. AFP-negative was confirmed in 18 of these patients, composed of the AFP-negative GC group (AFP[−]). The essential characteristics of these AFP[+] and AFP[−] patients are shown in Table 1. GC specimens from both groups were subjected to c-Met staining. All patients were staged according to the tumor, node, and metastasis (TNM) staging of GC, AJCC, 7th edition, 2010 (Table 2).

**Table 1:** Basic characteristics of patients

	Gender	Age	Disease condition	
**AFP [+]**				
1	F	56	Ulcerative poorly differentiated adenocarcinoma, some of which are signet ring cell carcinoma	T4aN1M0
2	F	77	Ulcerative adenocarcinoma	T4aN2M0
3	M	67	Invasive adenocarcinoma, a small amount of mucinous adenocarcinoma	T4aN2M0
4	M	45	Gastric infiltrating poorly differentiated adenocarcinoma	T3N3bM1
5	F	59	Invasive poorly differentiated adenocarcinoma, some of which are signet ring cell carcinoma	T4aN3aM0
6	F	72	Ulcerative adenocarcinoma	T4bN3aM0
7	M	60	Ulcerative adenocarcinoma	T4aN0M0
8	M	54	Ulcerative adenocarcinoma	T4aN1M0
9	M	50	Ulcerative adenocarcinoma	T3N1M0
10	M	80	Ulcerative adenocarcinoma	T4aN0M0
11	M	71	Ulcerative adenocarcinoma	T4aN2M0
12	F	75	Ulcerative adenocarcinoma	T2N1M0
13	M	64	Ulcerative adenocarcinoma	T4bN2M0
14	M	54	Ulcerative adenocarcinoma	T4aN1M0
15	M	60	Ulcerative adenocarcinoma	T4aN3bM0
16	M	73	Ulcerative adenocarcinoma	T4aN3aM1
17	M	59	Ulcer and elevated adenocarcinoma	T4bN3bM0
18	M	62	Superficial ulcerative adenocarcinoma	T1bN3aM0
19	M	38	Mushroom adenocarcinoma	T4aN0M0
20	M	61	Ulcerative adenocarcinoma	T4aN1M0
21	M	60	Ulcerative adenocarcinoma	T4aN1M0
22	M	65	Hepatoid adenocarcinoma, partly mucinous adenocarcinoma	T4bN0M0
23	M	75	Ulcerative adenocarcinoma, partly differentiated towards neuroendocrine	T4aN3aM0
**AFP [−]**				
1	M	62	Ulcerative adenocarcinoma	T4aN3aM0
2	M	75	Ulcerative adenocarcinoma	T4bN3aM0
3	M	49	Mucinous adenocarcinoma	T4aN0M0
4	F	58	Ulcerative adenocarcinoma	T3N1M0
5	M	55	Ulcerative adenocarcinoma, partly mucinous adenocarcinoma	T4aN0M0
6	M	69	Ulcerative poorly differentiated adenocarcinoma	T4bN3bM0
7	M	72	Ulcerative adenocarcinoma	T1bN3aM0
8	F	73	Ulcerative adenocarcinoma	T4aN0M0
9	M	63	Ulcerative adenocarcinoma	T4aN1M0
10	M	67	Ulcerative poorly differentiated adenocarcinoma	T4bN0M0
11	F	54	Ulcerative adenocarcinoma	T4aN3aM0
12	F	74	Mucinous adenocarcinoma, partial seal ring	T4aN1M0
13	M	59	Ulcerative adenocarcinoma	T4aN2M0
14	M	62	Ulcerative adenocarcinoma	T4aN2M0
15	M	75	Ulcerative adenocarcinoma	T3N3bM1
16	F	77	Ulcerative adenocarcinoma	T4aN3aM0
17	M	47	Ulcerative adenocarcinoma	T4bN3aM0
18	F	80	Ulcerative adenocarcinoma	T4aN1M0

**Table 2:** Comparison of the c-Met expression in gastric carcinomas of the AFP(+) and AFP(−) groups

	AFP (+)	*N* = 23		AFP (−)	*N* = 18		*p* value
Stage	−	+	++	−	+	++	
I	0	0	0	1	1	0	
II	0	2	4	1	4	1	
III	4	4	7	2	7	1	
IV	0	1	1	0	0	0	
	4	7	12	4	12	2	<0.01

All patients were staged according to TNM staging of GC, AJCC, 7th edition, 2010. Significance according to the *χ* ^2^ test.


**Informed consent:** Informed consent has been obtained from all individuals included in this study.
**Ethical approval:** The research related to human use has been complied with all the relevant national regulations, institutional policies, and in accordance with the tenets of the Helsinki Declaration, and has been approved by the Medical Ethics Committee of Huashan Hospital, Fudan University (ethical number: #2018-280).

### 2.2 Inclusion and exclusion criteria

Subject inclusion criteria were as follows: (i) age from 18 to 80 years; (ii) the preoperative clinical diagnosis was a malignant gastric tumor, (iii) the tumor could be resected locally by preoperative assessment, (iv) the postoperative pathological specimen was more than 1 cm × 1 cm, (v) the patient chooses to undergo surgery first, and (vi) AFP-positive blood test and immunohistochemical staining of pathological specimens were included in the AFP[+] group; otherwise, they were included in the AFP[−] group.

Subject exclusion criteria were as follows: (i) postoperative pathological non-adenocarcinoma, (ii) the patient was suffering from other malignant tumors at the same time, (iii) pregnant patients, and (iv) patients already participated in other research.

### 2.3 Immunohistochemical staining and analysis

**Figure A1:**
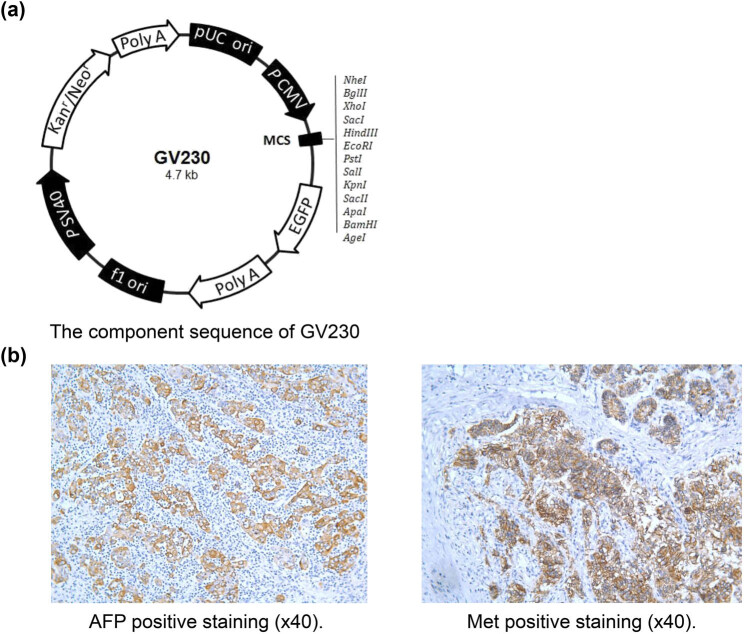
GV230 component sequence, AFP, and Met staining. (a) The component sequence of GV230. (b) AFP-positive staining (40×). Met-positive staining (40×).

Immunohistochemical staining was performed using the streptavidin–biotin–peroxidase method [19]. Briefly, the sections were deparaffinized and rehydrated, followed by 3% hydrogen peroxide incubation and non-specific antibody-binding site blocking. Then, the sections were incubated overnight at 20–25°C with a 1:50 dilution of the primary antibodies – AFP and Met (diluted 1:1,000; Cell Signaling Technology, MA, USA). The anti-AFP antibody was a rabbit monoclonal antibody to human AFP (Invitrogen, No. 37-0100, D12C1, Rabbit mAb). The anti-Met antibody was a rabbit monoclonal antibody to human Met (Invitrogen, No. 14-6499-82, D1C2, XP^®^, Rabbit mAb). The following day, the sections were washed in phosphate buffered saline (PBS) and incubated at 20–25°C with biotinylated secondary antibodies. The sections were incubated with diaminobenzidine to visualize the antigens and afterward counterstained with hematoxylin, dehydrated, and mounted. Negative control sections were treated with PBS instead of the primary antibodies, and AFP- and Met-positive liver cancer sections were used as a positive control. All sections were classified according to the grade of immunostaining in the carcinoma cells: negative (−), no carcinoma cells were stained; moderate positive (+), less than two-thirds of the cells were stained; and strong positive (++), more than two-thirds of the cells were stained (Figure A1(b)).

### 2.4 Cell culture and grouping

The AFP-GC cell line GCIY purchased from RIKEN BioResource Center, Japan, was cultured in a minimum essential medium containing 15% fetal bovine serum (FBS) at 37°C in a 5% CO_2_ atmosphere. Cells after transfection were cultured in Dulbecco’s modified Eagle’s medium (DMEM) with 10% FBS.

Cells were divided into the following groups: blank control group, GV230 group (vector GV230 with no AFP gene was transfected into the cells), GV230-AFP group (vector GV230-AFP was transfected into the cells), Met esiRNA group (Met esiRNA was transfected into the cells), GV230-AFP + Met esiRNA group (both GV230-AFP and Met esiRNA were transfected into the cells), NC esiRNA group (negative control esiRNA was transfected into the cells), and AFP esiRNA group (AFP esiRNA was transfected into the cells).

### 2.5 Cell transfection

AFP and Met endoribonuclease-prepared siRNA (esiRNA) were purchased from Sigma-Aldrich (Missouri, USA). esiRNA is a complex mixture of siRNA-like molecules prepared by enzymatic digestion of a long dsRNA molecule transcribed by an RNase III *in vitro* [20]. Lipofectamine^®^ RNAiMAX (Invitrogen) was used as a transfection reagent. About 50 ng of each kind of esiRNA in 5 μL TE buffer was diluted with 0.05 μL Lipofectamine^®^ RNAiMAX in 5 μL Opti-MEM (Gibco, USA) in a 24-well tissue culture plate. This reaction was then incubated for 20 min at room temperature. Wells containing the transfection reagent only were also prepared as the control group. As for AFP overexpression (GV230-AFP), the vector CMV-MCS-EGFP-SV40-Neomycin shown in Figure A1(a) was purchased from GeneChem (Shanghai, China) with the insertion sites XhoI/KpnI. The polymerase chain reaction (PCR) products of AFP were identified by gene sequencing and confirmed for uniformity with the human AFP gene sequence. For transfection, 0.8 μg GV230-AFP in 100 μL DMEM was diluted with 0.4 μL Lipofectamine^®^ 2000 in 100 μL DMEM in a 24-well tissue culture plate and then incubated for 20 min at room temperature. For esiRNA and GV230-AFP co-transfection, Lipofectamine^®^ 3000 (Invitrogen) was used as the transfection reagent to achieve better transfection efficacy.

### 2.6 Cell counting kit-8 (CCK-8) cell proliferation assay

Cells were plated in a 96-well plate at a density of 0.5 × 10^4^ cells/well and cultured overnight. Cells were transfected with the indicated overexpression vector and/or esiRNA and incubated for 24, 48, and 72 h, respectively. The CCK-8 (Beyotime, Hangzhou, China) was performed to determine the cell viability after transfection. CCK-8 reagent of 10 μL was added to each well at 1 h before the endpoint of incubation. Absorbance at 450 nm was measured using an automatic microplate reader (RNE90002, Reagen Biology LLC, USA).

### 2.7 Cell invasion and migration assay

For cell migration assay, 1 × 10^5^ cells in 100 µL of serum-free medium were added in the upper Transwell chamber (8.0-µm pore size; Corning, NY, CA, USA). For the invasion assay, the upper chamber was coated with Matrigel (1:10; BD Biosciences, MA, USA). Medium containing 10% FBS was added to the lower chambers. After migrating or invading for 48 h, the cells at the lower surface were fixed with methanol and stained with 0.1% crystal violet. Cells were counted, and images were acquired using an Olympus BX43 Motorized Microscope at a magnification of 100×. The experiments were repeated three times.

### 2.8 Total RNA extraction and qRT-PCR

Total RNAs of cells from blank control, GV230, GV230-AFP group, NC esiRNA, and AFP esiRNA group were isolated using TRIzol^®^ reagent according to the standard RNA isolation protocol. According to the manufacturer’s protocol, cDNA synthesis was performed with SuperScript™ III Reverse Transcriptase (Invitrogen). The primers used were as follows. AFP: forward 5′–GCAGAGGAGATGTGCTGGATTG–3′, reverse 5′–CGTGGTCAGTTTGCAGCATTCTG–3′; Met: forward 5′–TGCACAGTTGGTCCTGCCATGA–3′, reverse 5–CAGCCATAGGACCGTATTTCGG–3. GAPDH: forward 5′ GGTGAAGGTCGGAGTCAACG–3′; reverse 5′ CAAAGTTGTCATGGATGHACC–3′ and β-actin: forward 5′ CACCATTGGCAATGAGCGGTTC–3′; reverse 5′ AGGTCTTTGCGGATGTCCACGT–3′. GAPDH and β-actin were used as internal references. The relative quantification of messenger RNA (mRNA) expression was calculated using the 2^−∆∆Ct^ method.

### 2.9 Western blotting

Cells from the blank control group, GV230 group, and GV230-AFP group were harvested and lysed with RIPA buffer (Beyotime, Shanghai, China, No. P0013B). Protein concentrations were measured using a BCA protein quantification kit (Sigma-Aldrich, No. 71285-3). Protein samples of 50 µg were separated on 10% SDS gel and transferred to a polyvinylidene fluoride membrane, followed by 1 h of blocking with 5% skim milk. The membrane was then incubated with primary antibody of c-Met (Invitrogen, No. 37-0100), AFP (Invitrogen, No. 14-6499-82), and GAPDH (Invitrogen, No. 39-8600) overnight at 4°C, followed by three washes with Tris-buffered saline containing 0.1% Tween-20 (TBST). The membrane was then incubated with a horseradish peroxidase-conjugated secondary antibody HRP-labeled Goat Anti-Mouse IgG (H + L) (1:1,000, Beyotime, Shanghai, China; Cat. No. A0216) for 1 h at room temperature. After three washes with TBST, each single protein band of western blot was visualized using an enhanced chemiluminescence reagent and analyzed using ImageJ.

### 2.10 Statistical analysis

Data are presented as a mean ± standard deviation (SD), and the significance level was calculated according to the χ2 test. *p* < 0.5 was considered statistically significant.

## 3 Results

### 3.1 c-Met-positive expression in the AFP groups

**Figure 1:**
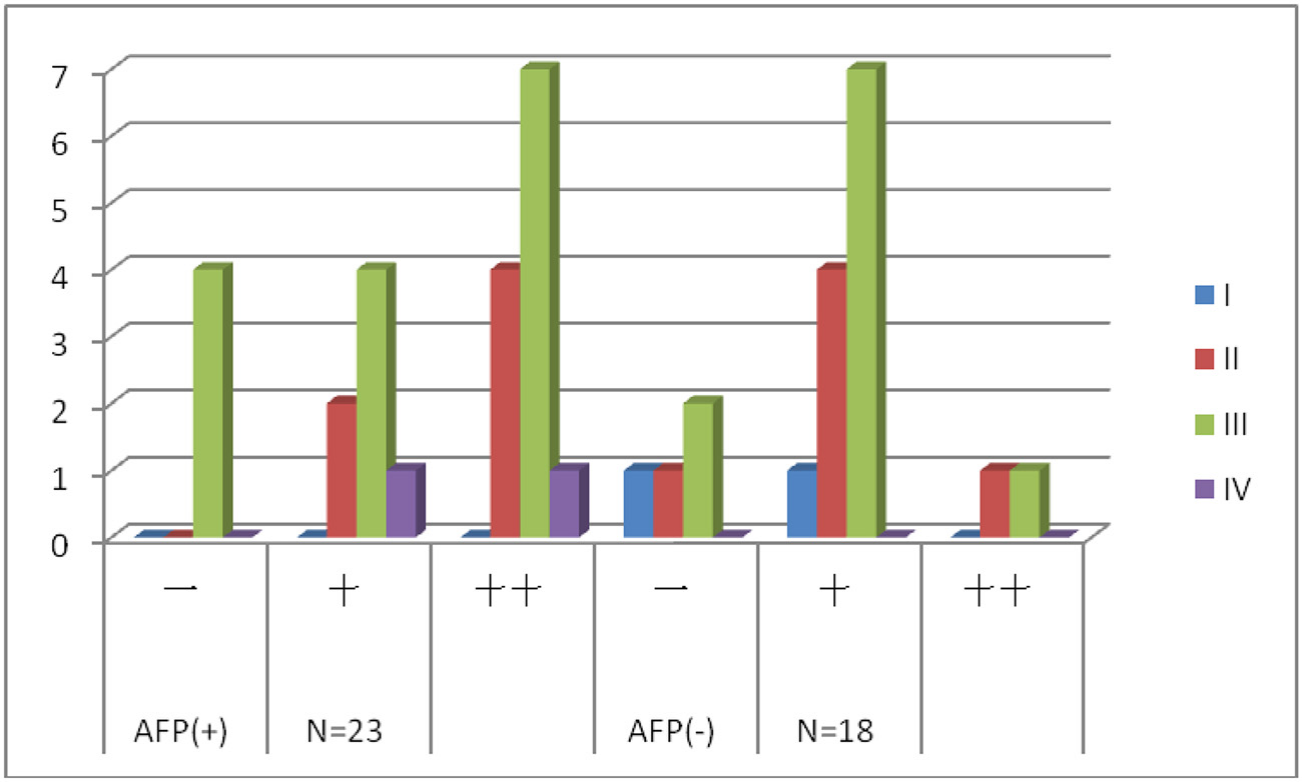
C-Met expression in gastric carcinomas in the AFP(+) and AFP(−) groups. All patients were staged according to TNM staging of GC, AJCC, 7th edition, 2010.

As shown in Figure 1, the immunohistochemical analysis in our study revealed that the overall incidence of c-Met-positive expression in the AFP[+] group was 19/23 (82.5%), in which strong positive incidence was 12/23 (52.2%). At the same time, the overall incidence of c-Met-positive expression in the AFP[−] group was 14/18 (77.7%), but the strong positive incidence was relatively low, which was 2/18 (11.1%) (*p* < 0.01). However, the differences in the overall incidence of the c-Met expression between the two groups were not statistically significant (*p* > 0.05).

### 3.2 Met played an essential role in AFP-induced proliferation in AFP-GC

**Figure 2:**
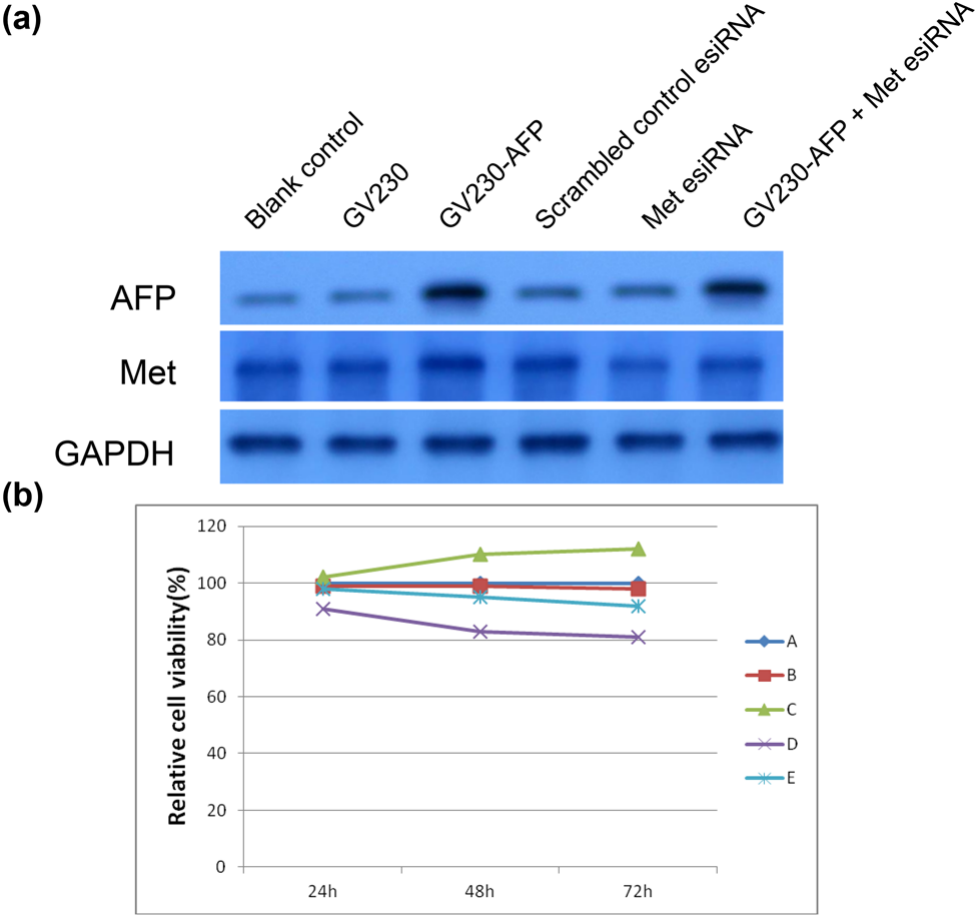
CCK-8 cell proliferation assay results. a: The efficacy of AFP overexpression and Met knockdown. b: Comparison between a blank control group and the AFP overexpression/Met knockdown group cell numbers. b: (A) Blank control group, (B) GV230 group, (C) GV230-AFP group, (D) Met esiRNA group, and (E) GV230-AFP + Met esiRNA group.

To elucidate the phenotype effect of AFP and c-Met in AFP-GC, we first established AFP overexpression and Met knockdown cell lines. The transfection efficacy is shown in Figure 2a. Moreover, corresponding to the result of Figure 1, the cell numbers after transfection for 48 and 72 h increased significantly in the AFP overexpression group, which showed a significant decline in the Met knockdown group. Interestingly, the AFP overexpression/Met knockdown group’s cell numbers showed no significant difference from the blank control group. These results indicate that while AFP could affect the proliferation of AFP-GC, Met may be a critical factor (Figure 2b).

### 3.3 Effect of AFP and Met on the invasion and migration of AFP-GC

**Figure 3:**
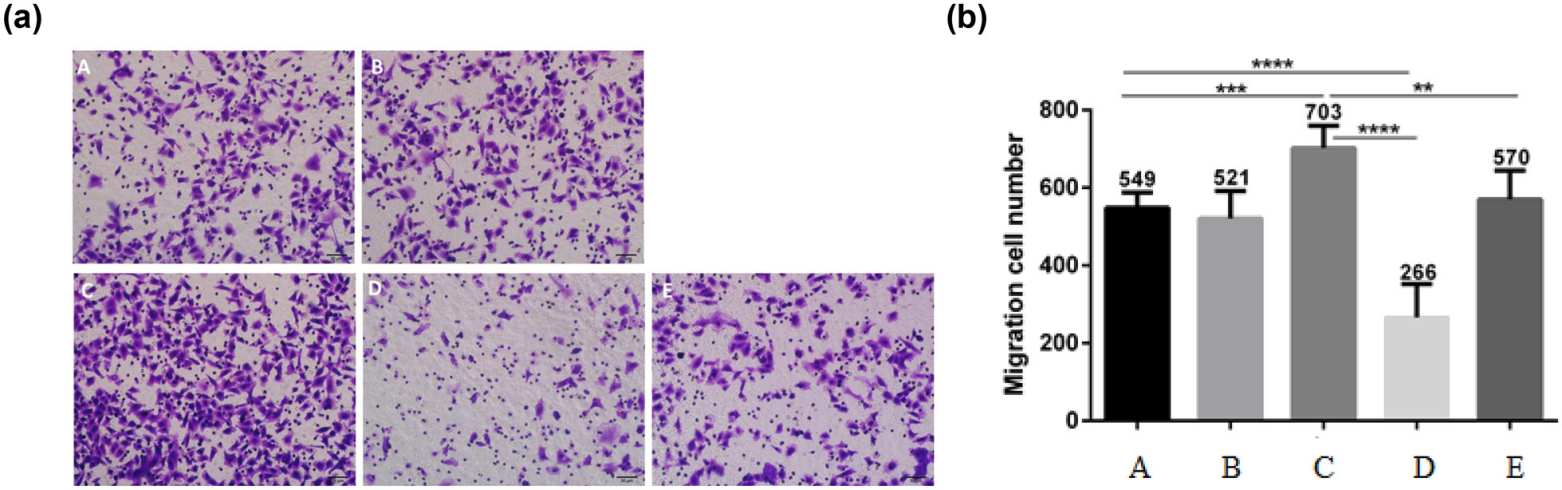
Cell migration assay results. a: (A) Blank control group, (B) GV230 group, (C) GV230-AFP group, (D) Met siRNA group, and (E) GV230-AFP + Met siRNA group. b: Migration cell number.

**Figure 4:**
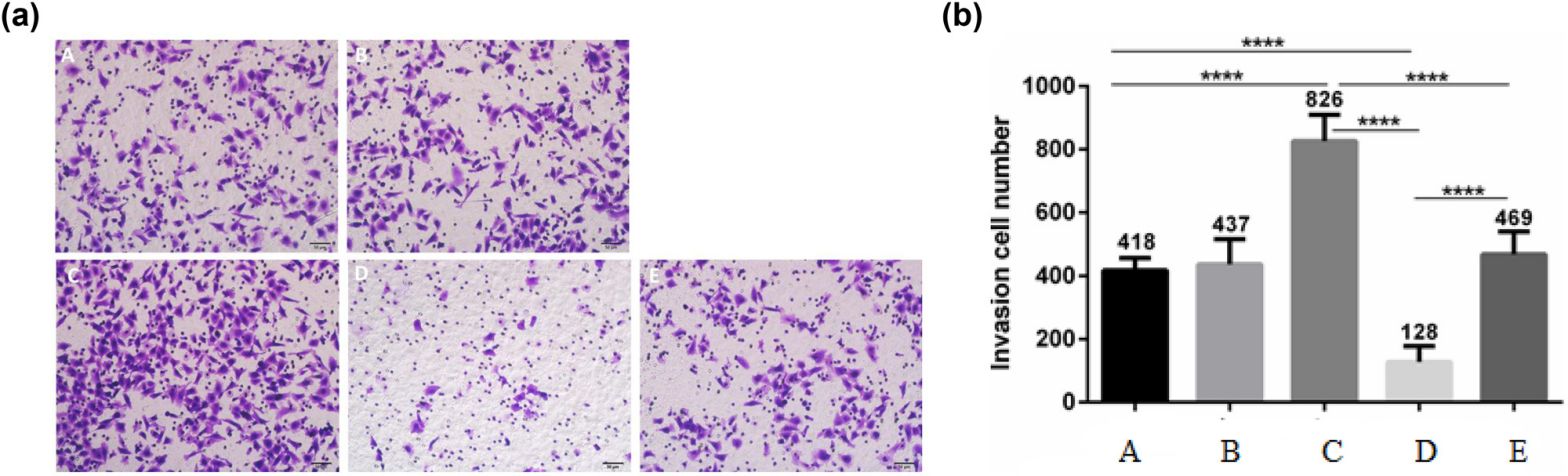
Cell invasion assay results. a: (A) Blank control group, (B) GV230 group, (C) GV230-AFP group, (D) Met esiRNA group, and (E) GV230-AFP + Met esiRNA group. b: Invasion cell number.

Furthermore, Figures 3 and 4 show that the AFP overexpression enhanced the invasion and migration of GCIY cells, while Met knockdown showed the opposite effects. Simultaneously, the AFP overexpression/Met knockdown group showed no significant difference from the blank control group. These results further confirmed that while AFP could also affect the invasion and migration, Met may play an essential role in AFP-GC progressions.

### 3.4 AFP positively correlated with the c-Met expression

**Figure 5:**
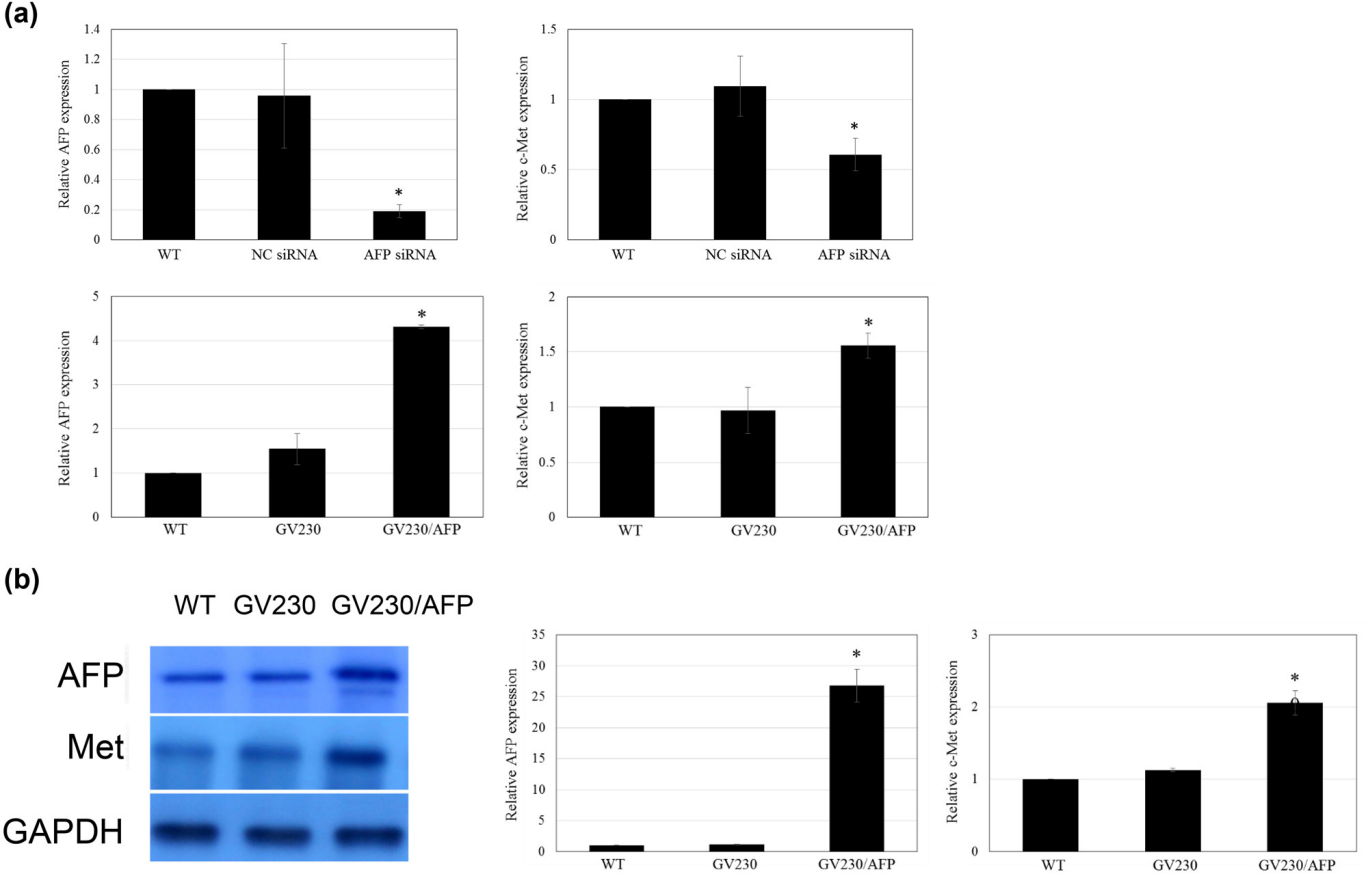
The relationship between AFP and Met. a: Knockdown of AFP reduces the c-Met expression, while the AFP overexpression in GCIY human GC cells elevates the c-Met expression. The AFP-siRNA reduces the expression of c-Met. The c-Met expression is significantly increased when AFP is overexpressed. b: Western blot showing overexpression of AFP in GCIY cells markedly induced the expression of c-Met in comparison with cells transfected with an empty GV230 vector.

To explore the potential relationship between AFP and c-Met expression in AFP-GC, both qRT-PCR and western blot analysis were used to detect either AFP or c-Met expression levels in the AFP overexpression/knockdown GCIY cell lines. Figure 5a demonstrates that AFP esiRNA achieved a knockdown efficiency of approximately 80% and 40% in the c-Met expression at the mRNA level, showing the positive correlation between AFP and Met in GCIY cells. Correspondingly, western blot results showed that the AFP overexpression led to the upregulation of Met at protein levels in human GC cells (Figure 5b).

## 4 Discussion

AFP-GC is a distinct type of GC, in which AFP can be tested in patients’ serum and/or cancer cells. A higher positive incidence of the c-Met expression was found in our study’s AFP[+] group. The frequency of the c-Met expression was increased by overexpression, whereas it was decreased by knockdown of AFP. In addition, our study also revealed that the AFP could affect the malignant biological properties of AFP-GC.

AFP-GC was first reported by Bourreille et al. in 1973. However, AFP-GC with hepatoid differentiation was described for the first time by Ooi et al. in 1985 and named “hepatoid gastric cancer (hepatoid GC)” [7]. This hepatoid GC and AFP-GC exhibit a high frequency of vascular invasion, lymph node and liver metastasis, and poor prognosis. A recent study found that liver metastasis in patients with hepatoid GC is 75.6%, and 1-, 3-, and 5-year survival rates are 30, 13, and 9%, respectively. The study also revealed that the liver metastasis rate of AFP-GC patients without hepatoid differentiation is 49.2%, and 1-, 3-, and 5-year survival rates are 64, 47, and 41%, respectively. Furthermore, the rate of liver metastasis in patients with typical GC is 11.5%, and 1-, 3-, and 5-year survival rates are 95, 57, and 38%, respectively [21]. Therefore, AFP-GC is associated with a higher incidence of liver metastasis and a lower survival rate than typical GC, even without hepatoid differentiation.

Correspondingly, the high frequency of liver metastasis in AFP-GC may be linked with overexpression of c-Met, a receptor of HGF and encoded by the c-Met proto-oncogene. Gardner et al., in their study, have shown that the expression of c-Met can enhance the ability of metastatic liver melanomas [22]. Subsequently, Krause et al. proved that the c-Met pathway is related to liver metastasis of colon cancer. They found that a higher frequency of the c-Met expression leads to a higher recurrence rate after resectioning of metastatic liver cancer [23]. Lee et al. also indicated that the c-Met expression in metastatic liver GC is much higher than in the primary cancer [24]. Another study reported a higher c-Met expression level in AFP-GC than in GCs that do not express AFP [18]. The overall positive incidence of c-Met in common GCs ranges from 18% to 71.1%. In addition, gene amplification of c-Met is correlated with cancer stages, and overexpression of c-Met is noticed in GCs with deeper invasion and distant metastasis [25].

Nevertheless, the mechanism linking the c-Met expression and AFP remains uncertain. Our research revealed that there is no significant difference between the incidence of the c-Met expression in AFP-positive and AFP-negative GC. However, the strong positive incidence of the c-Met expression was much higher in AFP-positive GC than in AFP-negative GC, reinforcing the previous studies.

While the Met pathway plays a critical role in cancer cells’ invasion and metastasis abilities, the Met proto-oncogene encodes a transmembrane receptor–protein tyrosine kinase. Overexpression of this transmembrane receptor is associated with poor prognosis in various cancers [22]. On the other hand, knockdown and overexpression of AFP can cause a change in the Met expression, which implies that AFP can affect the expression of c-Met through an unknown pathway. Apart from this, AFP may directly play a role in c-Met as a transcription factor or regulate the expression via other transcription factors. However, all these assumptions need further exploration. Therefore, an in-depth understanding of how AFP regulates Met may be essential for developing therapeutics to treat AFP-GC.

Furthermore, the results of this study may indicate that AFP regulates the expression of c-Met, and the effect might be direct. It would require the translocation of AFP to the nucleus or indirectly through the action of AFP on one or more transcription factors that regulate c-Met directly. However, more studies are needed for further exploration of these assumptions. For instance, recent reports have demonstrated that AFP may function as a regulator of the phosphatidylinositol 3-kinase/Akt pathway hepatocellular carcinoma cells in humans [26,27]. They found that transfection of AFP-cDNA into hepatoma HLE cells (originally AFP-negative) led to a significant activation of the Akt signaling pathway [26]. Another study found that transcription factor protein 1 (Sp1) and mothers against decapentaplegic homolog 3 (Smad3) mediate the c-Met expression in renal epithelial cells [28]. Moreover, an increased expression of c-Met is associated with the upregulation of hypoxia-inducible factor-1 (HIF-1) in tumor cells, especially in papillary carcinoma of the thyroid [29]. Despite this, multiple studies reported the regulation of Sp1 and HIF-1 expression and Smad3 phosphorylation by the Akt signaling pathway [30,31,32].

Taken together, a better understanding of the biological activities of AFP as a growth regulatory cell-signaling factor has emerged [13]. This study revealed that AFP could affect the malignant behavior properties of AFP-GC. However, according to a recent study, there are several controversies regarding the clinicopathologic and prognostic features of the AFP-GC [15]. Another recent study suggested that a significant decline in the serum AFP level was found to be associated with good treatment response and prognosis of AFP-GC. Moreover, TNM staging classification stage, liver metastasis, and curable surgery were also noticed to be associated with prognosis in their observational study [33]. Therefore, this provides new insight for further studies considering AFP affects the malignant behavior’s properties in AFP-GC and thereby with a possible decline in the serum AFP level. These demonstrate that future research on this topic will benefit from finding the potential therapeutic targets/adjunct therapy due to the relevance of AFP for patients with AFP-GC.

This study also has several limitations. (i) This study does not provide the detailed mechanisms by which AFP affects the c-Met expression. At this stage, some hypotheses can only be made and require further exploration through studies. (ii) Some of the SDs of this study seem high. (iii) Furthermore, some figures do not show all data points and proper error bars. However, additional experiments/results could not be provided due to the ongoing pandemic situation in China and will consider in future studies.

In conclusion, based on these studies, we suggest that AFP might regulate the expression of c-Met through the activation of the Akt pathway. We plan to investigate this hypothesis in the future.
